# Who gets the gametes? An argument for a points system for fertility patients

**DOI:** 10.1111/bioe.12411

**Published:** 2017-12-01

**Authors:** Simon Jenkins, Jonathan Ives, Sue Avery, Heather Draper

**Keywords:** allocation, eggs, fertility, gametes, points, reproduction, sperm

## Abstract

This paper argues that the convention of allocating donated gametes on a ‘first come, first served’ basis should be replaced with an allocation system that takes into account more morally relevant criteria than waiting time. This conclusion was developed using an empirical bioethics methodology, which involved a study of the views of 18 staff members from seven U.K. fertility clinics, and 20 academics, policy‐makers, representatives of patient groups, and other relevant professionals, on the allocation of donated sperm and eggs. Against these views, we consider some nuanced ways of including criteria in a points allocation system. We argue that such a system is more ethically robust than ‘first come, first served’, but we acknowledge that our results suggest that a points system will meet with resistance from those working in the field. We conclude that criteria such as a patient's age, potentially damaging substance use, and parental status should be used to allocate points and determine which patients receive treatment and in what order. These and other factors should be applied according to how they bear on considerations like child welfare, patient welfare, and the effectiveness of the proposed treatment.

## 1 INTRODUCTION

This paper addresses gamete allocation in any country where allocation is not determined by market forces (where gametes go to the highest bidder). Donated gametes are a scarce resource.^1^ Given such scarcity, not all patients who wait for treatment will be treated; as the waiting list continues to expand, some waiting patients will become too old to be treated effectively. An alternative system is to waitlist only as many patients as there are likely to be gametes available, using morally robust criteria for prioritizing those selected. Notwithstanding questions of resource scarcity, gamete allocation at the level of individuals requires ethical judgement. For instance, treatment may not serve the overall interests of the patient. The welfare of any resulting child(ren) is also an important ethical consideration, sometimes reinforced in law,^2^ regardless of whether or not *donated* gametes are used. Hence, the ethical issues discussed here reflect concerns about assisted reproduction in general. Donated gametes present additional challenges because resource scarcity means that some of those seeking treatment will be disappointed. Allocation must, therefore, be based on appropriate, fair, and transparent criteria.

We discuss some of the key ethical issues surrounding gamete allocation with reference to a qualitative study that explored the views and practices of U.K. fertility practitioners and other relevant parties (e.g., academics and representatives of patient groups). These views indicate the extent to which philosophical recommendations about gamete allocation might be received in practice, enabling modifications to increase their acceptability to policy‐makers. The principal recommendation that will be argued for in this paper is that patients should not be treated solely in date order, but rather that other, morally relevant, criteria should be given more weight in deciding who to treat and when. We propose a points system for gamete allocation, and discuss age and potentially damaging substance use as examples of morally relevant criteria for prioritization. These examples will help to show how the system could operate.

## 2 METHODS

Our study design was informed by empirical bioethics methodology, which attempts to ensure that normative ethical analysis is context‐sensitive and grounded in the realities of day‐to‐day practice.^3^ Having ‘encounters with experience’^4^ means that important features of the practice under investigation are identified from the everyday lived experience of practitioners and stakeholders and given serious consideration. The approach gives weight to Mill's view that:

In the case of any person whose judgement is really deserving of confidence, how has it become so? […] Because he has felt, that the only way in which a human being can make some approach to knowing the whole of a subject, is by hearing what can be said about it by persons of every variety of opinion, and studying all modes in which it can be looked at by every character of mind^5^

Taking this approach opens up avenues for principled and justified compromise^6^ that may be a necessary part of any serious practical normative proposal.

**Figure 1:**
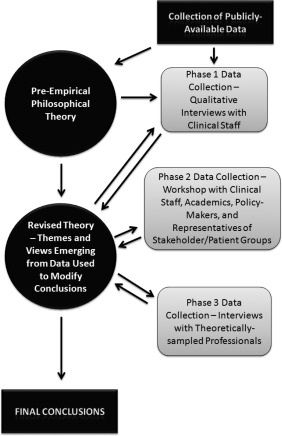
Research process

A review and discussion of existing relevant philosophical literature generated a set of 11 initial conclusions. These conclusions were then developed in light of primary interview data that provided contextualized criticism or support of those conclusions. The revised conclusions were then presented to a wider stakeholder group (followed up with selected interviews) and a final round of revision (see Figure 1). This iterative process of forming initial conclusions and then exposing them to systematic challenge through empirical encounters is reminiscent of the reflexive balancing methodology outlined by Ives^7^: the conclusions were revised through this process. This paper focuses on just one of these revised conclusions, the argument for which is outlined below (see ‘The Basic Proposal – Points and Exclusion’). Other conclusions included recommendations about which criteria should be prioritized and how. Given their number and complexity there is not scope to discuss them all in this paper, but some of them will be used here to explore how the main proposal could work.

Encounters with experience for this project were gained during the three data collection phases (see Table 1), and were designed to develop normative conclusions iteratively, providing the opportunity for the emergent theory to be modified by these encounters, and allowing the shape of the encounters to be informed by the emerging theory.

**Table 1:** Data collection phases and participants

			Composition		
Phase	Type	Participants	Roles	Genders	Clinic Information (n = 7)
1	Face‐to‐face interviews lasting 45–68 min (mean average 55 min)	Fertility clinic staff (n = 18)	3 egg donor co‐ordinators; 3 nursing staff members; 2 sperm donor co‐ordinators; 2 medical directors; 2 counsellors; 2 gynaecologists; 1 clinical midwife; 1 embryologist; 2 other clinical staff	14 female; 4 male	3 in Scotland; 3 in England; 1 in Wales 5 NHS operated; 2 privately operated
2	Workshop (5.5 hr)	Practitioners, academics, policy‐makers, representatives of patient groups, and other relevant professionals (n = 20)	11 fertility clinic staff members (8 from phase 1); 3 academics; and representatives of: the Human Fertilisation and Embryology Authority (HFEA) (2 participants); the National Gamete Donation Trust (NGDT); Progress Educational Trust (PET); the Donor Conception Network (DCN); and the British Medical Association Ethics Committee	15 female; 5 male	N/A
3	Telephone interviews lasting 39–48 min (mean average 44 min) and one email ‘interview’	Academics and representatives of patient groups (n = 3)	2 from phase 2; 1 new participant^10^	2 female; 1 male	N/A

Phase one made use of purposive sampling for maximum variation so that a broad range of clinics was represented. Clinics were selected from the list of all Human Fertilisation and Embryology Authority (HFEA)‐licensed clinics on the HFEA website. Difficulties obtaining timely local permissions^8^ meant that timely recruitment from Northern Ireland within the study period was not possible.

A limited snowballing approach^9^ to recruitment was also used. When being invited to the study, staff members were encouraged to pass recruitment information to colleagues who had a role in gamete allocation. Self‐selection of participants by clinics was inevitable. Not all clinics employed staff with a clearly defined responsibility for coordinating the allocation of donated gametes.

At phase two, participants were purposively sampled to include a range of disciplinary, professional, and lay perspectives. Twenty participants took part.

Phase three employed purposive and theoretical sampling. Only those who were identified as potentially making a detailed and important contribution to specific issues were included, and this explains the small sample. One participant, a representative of a patient group, was unable to attend the workshop. The other two participants had attended and were theoretically sampled on the basis that the views they had expressed presented specific challenges to the emerging theory that required further detailed exploration.

Data from the interviews and workshop were transcribed verbatim, and the transcripts were coded using NVivo ([author 1]) ‘to facilitate an accurate and transparent data analysis process.’^11^ The data underwent thematic analysis using the process described by Braun and Clarke, and included the following steps: familiarization with the data through transcription, re‐reading, and annotation of transcripts with initial ideas; generation of codes and themes; and finally, generation of visual thematic maps to demonstrate the relationships between themes.^12^

To enhance trustworthiness of the analysis, a sample of coded transcripts was independently checked by other members of the team ([author 4] and [author 2]), which ensured the analysis was not dominated a single researcher perspective or agenda. Themes emerging from the data were discussed and agreed on by all authors. Interviews were conducted concurrently with these analysis discussions. As such, the processes of data collection and analysis in phase one were iterative (as is consistent with the overall empirical bioethics methodology adopted), with the analysis of earlier interviews affecting what was explored in later ones. The data analysis was thus partly directed and partly conventional,^13^ as it was both theoretically‐ and data‐driven.

Phase two and three data analysis was more theory‐driven. Themes and important issues had already been established, both by a philosophical analysis undertaken before data collection, and by phase one data analysis. By this stage the analysis focussed on fine‐tuning particular concepts, so the coding was more directed (see Figure 1).

The project received a favourable research ethics review (reference 11/WM/0099), and appropriate local permissions to access participants were gained.

## 3 RESULTS AND DISCUSSION

The principal conclusion of the 11 conclusions generated by the initial philosophical work for this study was to replace the current ‘first come, first served’ (henceforth FCFS) allocation system for donated gametes with a points system. In this section, a normative argument will be presented and punctuated with data from our empirical ‘encounters with experience’.^14^ Accordingly, the narrative outlines the developing argument and draws on our empirical data to illustrate challenges and barriers that come from experience, which must be acknowledged and considered seriously. Putative criteria on which a points allocation system could be based are discussed. The prioritization criteria described all arose in the preliminary philosophical analysis, and were raised by our participants independently of prompting. This may suggest a fair degree of harmony in terms of the *identification* of moral issues.

### 3.1 The basic proposal—points and exclusion

The points‐based system is derived from Pennings' argument that this type of allocation ‘gives credit points to the *morally relevant* features of the prospective recipients’^15^ (emphasis added). These points, rather than the date of admission to the waiting list, are then used to determine who receives treatment. The number of patients treated may vary according to the gametes available, but patients with fewer points are less likely to get treatment at all. Those with a significantly low score should be effectively excluded because, in this system, they are in continual competition with everyone listed, including those listed later than them. Arguably, this is a fairer and more transparent system than one that allows people to ‘time expire’, which effectively excludes people on the more arbitrary grounds of when they applied for gametes. A significant benefit of a points system is that it ‘allows us to take into account more than one relevant factor simultaneously in the allocation procedure.’^16^ Those that are excluded, then, are excluded for better reasons than unfortunate timing.

A comparable system is already in place for publicly funded fertility treatment in New Zealand, with weighted criteria being used to allocate points, ‘reflecting the need and also the benefit obtained from ART [Assisted Reproductive Technology] treatment’.^17^ The model used is a threshold system, however, in which patients are treated on an FCFS basis once they reach a certain threshold. It is similar to our proposal in that it uses weighted criteria to allocate points, but different insofar as it does not distinguish between patients who reach the minimum number of points.

There is already much discussion of whether *any* criteria should be imposed as conditions for receiving fertility treatment, and of what those criteria should be.^18^ Some scholars argue that the fact that assisted reproduction is regulated and non‐assisted reproduction is not is an unjustifiable inequality,^19^ but others argue that at least some regulation of assisted reproduction, or even of reproduction generally, is justified.^20^ This paper will not address in detail the more general arguments for and against regulation. Rather, we begin with the assumption that *some* kind of regulation is justified, simply on the basis that there is morally relevant difference between natural and assisted reproduction such that in assisted reproduction there is a limited resource (donated gametes) that has to be managed and distributed. It is our view that it is best to debate allocation in a way that is divorced from issues related to control over access to infertility treatment per se. We think that it is coherent to hold liberal views about access per se to infertility treatment and against this background, to allocate scarce resources in as fair a way as possible, which requires regulation of some kind. The following discussion will refer to the participants' comments to draw out why we think the priority system is preferable to an FCFS system.

Our participants tended to think that once accepted for treatment, patients should receive gametes in the order of acceptance. Where prioritization based on other factors did not arise spontaneously in phase one interviews, participants were directly asked for their opinions. A points‐based system was presented at the workshop, with age and substance use^21^ proposed as examples of justifiable (de)prioritization criteria. The idea of prioritization on any other grounds than FCFS elicited mixed opinions. Some agreed that it could be acceptable in principle: ‘It seems to be unproblematic so long as the criteria for prioritisation are defensible’ (32^22^, p3^23^). Others were uncomfortable, and commonly invoked fairness or equality considerations as reasons against its use:

[W]ell I think everyone's sort of equal, just because we feel they've got a lower chance than someone that's 25 coming through who's probably gonna get pregnant first time, I don't think we can say they can't use them. I mean everyone should be entitled to use them equally (08, p1)

I find it difficult when you're saying about prioritising by age and having specifics that you would be excluded people from other than welfare of the child issues (04, p2)

Participant 08 here argues for equal access to these resources, and 04 is uncomfortable with prioritizing or excluding on any basis other than child welfare concerns. Participants tended to report that exclusion from treatment only occurred in extreme cases (such as where serious child welfare concerns were raised by substance use or violent behaviour), and that patients at their clinics were normally treated in the order in which they were referred: ‘I think patients understand the principle of referral date. They understand “referred on this date”, put on the list, then get treated’ (22, p2). Participants were therefore quite resistant to the proposal to prioritize patients on criteria other than waiting time, and instead favoured treating all patients on an FCFS basis, except in extreme cases where a patient could be prioritized or excluded entirely.

A FCFS system could be described as a points system in which patients accumulate points the longer they wait. But a points system need not include waiting time at all. Indeed, if waiting time is morally *irrelevant*, then the system not only *need* not, but also *should* not include waiting time. The FCFS system is therefore doubly problematic: as well as failing to account for relevant criteria, it gives sole weight to a criteria whose moral relevance is far from certain or clear, and certainly not overriding.

Time on the waiting list is not morally relevant in itself, but longer waiting times may increase the psychological burden on patients and diminish the prospects of effective treatment. The former would be morally relevant if the additional time waiting (time Y) demonstrably worsened the experience because of the time already waited (time X): each passing (say) month of extra time Y had a greater impact by virtue of time X. Nonetheless, until the negative impact of waiting time has been established by evidence, it should not be given the sole and overriding weight it currently enjoys.

One reason participants gave in support of FCFS was that it ensured equal access to treatment, and equality was highly valued. They were, however, willing in some circumstances (e.g., if one of the prospective parents has a history of child abuse) to exclude patients altogether from treatment. This suggests that for the study participants, considerations of equality become pertinent only once patients meet some minimum threshold for treatment. The threshold seemed to be based primarily on three factors: concern to prevent foreseeable and significant harms to any resulting child, the safety of the patient, and the effectiveness of the treatment (the likelihood of its success). Given that all three of these factors relate directly to child or patient welfare, they were not considered morally problematic. That said, our participants held different views about whether already being a parent kept patients below the minimum threshold. For some, this included an existing child. This suggests that, for these participants, the purpose of treatment was to provide patients with a parenting experience. This being so, fair allocation of *any* treatment means placing those without children ahead of those with children. There was, however, disagreement about how to define this parenting experience (see below).

Returning first to their equality‐related reasons for preferring an FCFS system (once the threshold was met), participants tended to interpret ‘equality’ as requiring that every patient is given an equal chance of *receiving treatment*, so that from the time they are placed on the list, they are treated just the same as everybody else. A FCFS system may satisfy this notion of equality, but not if some patients have a worse chance than others because of criteria such as an upper age limit, which they may reach before they are treated.

Giving everyone an equal chance of having treatment therefore preserves equality in one respect. Given, however, that the actual desideratum for patients is not just to have treatment, but to become parents, this conception of equality is inappropriate. Giving everyone an equal chance to become parents would look very different to giving everyone an equal chance to have treatment, as it would have to take into account each patient's prognosis for successful conception, implantation, and bringing to term of a pregnancy. This difference is demonstrated by the scenario outlined in Box 1. The study participants' emphasis on equal access to treatment, rather than equal access to a successful pregnancy, fails, therefore, to account for the value of treatment effectiveness,^24^ and appears incompatible with the primary reason for offering treatment in the first place: to provide a parenting experience.

Box 1 Patient A, if treated, is twice as likely as patient B to conceive and carry a pregnancy to term. Tossing a coin would give them an equal chance of receiving gametes, but would not give them an equal chance of becoming a parent**—**A's chances would still be twice as good as B's. To equalize their chances of having a baby, the coin would need to be weighted in favour of B every time gametes became available, to factor in the fact that she is less likely to be successful in her treatment with the gametes.

Therefore, the FCFS system as a means of treating patients equally may actually fail to account for morally relevant differences between them, and only appears fair for the perhaps strange reason that it ignores these differences. It is our contention that the FCFS system has intuitive appeal because it *appears* to be a system that is free of value judgements, but it only appears so because it permits morally relevant criteria to be ignored. Box 1 demonstrates that this lionizing of a thin conception of fairness as value‐free service ignores the actual purpose of fertility treatment, by focussing on giving equal access to treatment rather than equal chance of achieving the desired outcome: children. Yet predictions about chances of success can be made in a way that is supported by evidence and is therefore arguably more value‐free and fair.

Morally relevant and irrelevant criteria are, by definition, those that should and should not (respectively) be taken into account. An FCFS system uses one criterion (time spent waiting), which ignores all other, morally relevant criteria. It seems clear that although there may be strong resistance, on the grounds of equality, from practitioners and stakeholders, this resistance arguably relies on the flawed assumption that the best expression of equality lies in an FCFS system. This resistance may nevertheless introduce practical difficulties when trying to introduce a points system into policy. A way to make the system more palatable is to allow waiting time to be considered as one criterion in the points system. This would incorporate the view that waiting time is relevant, but allow for other criteria to be considered also. This may make for a better system than one that *only* includes waiting time.

We appreciate that there are practical difficulties in obtaining some of the measurements required to determine the points that should be allocated to patients. The degree and quality of parenting experience is one such example, as different individuals from diverse backgrounds may have very different conceptions of what constitutes a sufficiently good parenting experience to exclude one from listing. Nonetheless, in a context in which not everyone can be treated, we should aim to choose fairly.

The remainder of this discussion will focus on some of the other candidates for prioritization criteria, beyond waiting time. Of the putative criteria for a prioritization system, age and substance use proved least controversial to participants. As we will show in the following sections, discussion of those criteria provided examples of cases in which participants supported excluding patients from treatment, though there was variation on the finer details.

### 3.2 Possible criteria for prioritization

#### 3.2.1 Age

While many participants were reluctant to espouse age‐based patient *prioritization*, some were willing to *exclude* patients from treatment because of their age, citing potential challenges to the patient as a reason for this:

[A]s good as we all look these days, you're still 42 years old your organs are still 42 years old, so a pregnancy can be slightly more challenging than if you're 32 years old' (03, p1).

Caplan and Patrizio describe ‘the safety of pregnancy for older women’^25^ as a concern, and the participants found common ground with these authors in this respect. This is perhaps unsurprising, given their professional obligation to ‘make the care of [their] patient [their] first concern.’^26^ The Nursing and Midwifery Council's Code contains a similar position: ‘[y]ou put the interests of people using or needing nursing or midwifery services first. You make their care and safety your main concern.’^27^ Codes of conduct reflect the view that patient welfare is a morally relevant concern, and while a patient may be aware of any risks and willing to take them, given that these risks make successful pregnancy less likely it may nevertheless be better, in circumstances of scarcity, to treat someone for whom these risks are not a factor. Indeed, these risks make a successful pregnancy less likely, so the scarcity of resources is relevant once again.

The use of donor eggs mitigates effectiveness concerns associated with female recipients' age.^28^ They may also reduce the risk of chromosomal abnormalities, which may improve child welfare, at least in some cases. Obstetric risks remain,^29^ however, which threaten the woman *and* the foetus/child.

The relationship between child welfare and maternal age becomes more complicated when we consider other measures of child welfare. Sutcliffe et al. found some evidence suggesting that children are better off as maternal age increases.^30^ Their study, which included children with mothers whose age ranged from 13 to 57, found that with increasing maternal age, children had ‘better language, and fewer social and emotional difficulties'.^31^ It only considered children up to the age of five, however, so the potential difficulties of losing one's mother at a younger age may be relevant. Many fertility clinics operate an upper age limit for *any* treatment (usually 40–50) for women, but this is not perceived to be part of prioritizing gamete allocation. This pre‐allocation threshold makes it unlikely that mothers will die before their children reach adulthood. The Sutcliffe et al. study also considered other health‐related issues, finding that children born to older mothers had fewer hospital admissions^32^ and greater levels of immunization. They did not, however, discuss chromosomal abnormalities (a risk which increases in older women when donor eggs are not being used) and other health problems at birth**—**indeed the study focussed on factors with environmental causes. Hence, some work must be done to weigh these different health risks against each other if age is to be used fairly in gamete allocation.

A simplified points system might be linked to data on the average severity of congenital conditions and the average severity of hospital admissions to arrive at an age threshold beyond which the statistical risks to the child were regarded as too great, although clinical information about individual cases may provide a reason to deviate from this threshold. This calculation would also need to evaluate and include the dangers of having fewer immunizations, and other relevant factors, and then weigh these up against the risks and harms of congenital conditions, to determine whether it is older or younger patients whose children can be expected to be healthier. In cases of egg donation, the age of the donor is relevant as well as the age of the patient. It is beyond the scope of this paper to undertake this calculation. Suffice it to say that as the Sutcliffe et al. study has not been reproduced, we should be less confident in this data than in the medical consensus on obstetric risks. Until further philosophical and empirical work has been done to compare these different potential harms to children, it is unclear whether the balance is tipped for or against older women.

A general objection to age as a prioritization criterion is that to take broader child welfare indicators into account opens the door to prioritizing based on *anything* that can affect child welfare, such as the parents' level of education or their socio‐economic status, which may result in damaging social inequalities. There may indeed be wider societal reasons to discount certain potential indicators**—**for example, if it were shown that there was a connection between race and child welfare due to social inequalities. Again considerations of equality emerge, but in a different form from those discussed above. It is at any rate our view that these factors must each be reviewed on a factor‐by‐factor basis. Child welfare remains a serious concern that should not be dismissed, and a commitment to prioritizing child welfare in circumstances of gamete scarcity does not commit one to arguing that it ought to be maximized in *all* other circumstances. The social harms of accounting for age may be different from the social harms of accounting for race. Our argument is that we should begin to have these discussions around each putative child welfare indicator, rather than pointing to uncomfortable factors as a reason to avoid using any criteria to allocate gametes, and thereby throwing the metaphorical baby out with the bathwater. Age is a relevant criterion, but how it should be accounted for will depend on a variety of factors relating to the effectiveness of treatment, and the welfare of parents and children. Age‐based criteria will need to be sensitive to these nuances in order to be fair.

Any criteria we choose will leave open the possibility of bias in the decision‐making process, simply because these criteria will be applied by humans. This is especially so if this decision‐making is left to individuals operating in isolation, who may apply these criteria according to their own values in a way that unjustly discriminates against certain patients. Our comment that each factor should be considered on a factor‐by‐factor basis should not be interpreted as our recommending that clinicians make decisions on these factors in isolation from a public discussion and agreement about how to proceed**—**in fact, exactly the opposite is true. Each putative social factor should be debated by the relevant policy‐makers and by those that generate guidance for clinical practice. A transparent process of implementing and overseeing the use of these criteria would help to ensure that these access criteria are applied in a fair and reasonable way. Notwithstanding the risk of bias in the application of any criteria, the purpose of this proposal is to make a call for morally relevant, evidence‐based criteria to be used for prioritization**—**which, we suggest, will be ultimately fairer that the current, single criterion FCFS system that allows for personal and non‐evidence‐based judgement when excluding on the basis of welfare of the child concerns.

#### 3.2.2 Substance use

It is widely agreed that using alcohol, tobacco, and certain illegal drugs (e.g., cannabis, ecstasy, cocaine, and heroin) while pregnant can have a negative impact on the foetus^33^ making it a strong candidate for a negative prioritization criterion on child welfare grounds. The HFEA acknowledges ‘drug or alcohol abuse’^34^ as considerations that are relevant to child welfare. The majority of study participants acknowledged child welfare as a major reason for being concerned about substance use:

[T]hey may be on drugs. Uh, various issues which would make it not in the welfare of the child to be, uh, of the a child born to them (09, p1)

Similar to drug abuse but certainly alcoholism um would also be a significant welfare of the child concern (10, p1)

We will now discuss how substance use could be factored into gamete allocation. One possibility is that the use of certain substances should exclude a patient outright, that is, operate as a threshold below which patients are not considered for treatment. Some of the study participants thought that anyone known to be using ‘recreational drugs’^35^ (14, p1) should not be offered fertility treatment:

[I]f people were, were using recreational drugs and had told us about it then again we would be looking for them to have stopped that before they were going through any treatment. (14, p1)

An emphasis on the use of *illegal* drugs as an exclusion criterion seems an untenable position, given that the teratogenic effects of different substances do not necessarily track their legality**—**alcohol may be worse in this respect than cannabis,^36^ for example. Furthermore, this policy would ignore the *extent* of the substance use**—**even if two substances are similar in terms of their effects on prospective children, extensive usage of a legal substance may be a weightier consideration than occasional usage of an illegal one.^37^

A course of action that would better account for such nuances would be for substance use not to exclude a patient, but for it to act as a spur to investigate that patient further. Some study participants reported such a policy:

[I]f any one member of staff feels there's a real reason to call into question treatment, they can call a meeting and ask for it all to be discussed. The final decision might not go their way. But at least it's been broadened it's been examined. To take things to that length we would ask for GPs' input, perhaps even depending if there'd been violence past social work input, if there'd been drugs in the past you know we'd ask for more input [Interviewer: yep] before we made any dramatic final decisions, either to treat them or to not treat them (05, p1)

As with age, then, the nuances of individual cases may determine how far substance use is used as a negative prioritization criterion.

An additional reason to de‐prioritize substance‐using patients is the increased risk of pregnancy complications and medical issues. This raises questions about effectiveness and patient welfare, which occasionally arose in our data, primarily with regard to smoking:

I would say smoking does affect health, isn't it and if the patients can't help themselves, why should somebody else help them? That's one. Smoking does affect fertility. Yeah. And if they don't want to stop it and help themselves, um, why should you fund a treatment which will be less accessible,^38^ because of their smoking? (16, p1)

In summary, substance use is linked to considerations about welfare of the child, and to concerns about patient welfare and treatment effectiveness. These features make it a relevant allocation criterion; denying access to donor gametes is not applied as a punitive measure or expression of disapproval. This is a more clear‐cut case than the age criterion in that it is more obvious that substance use should count against treatment, whereas it is unclear whether increased age should be used for or against treating a patient. However, there may be relevant differences in the type and degree of substance use, so it is therefore a shared feature of both of these criteria that finer‐grained differences between individual cases may give rise to different courses of action.

#### 3.2.3 Parental status

This may be taken to mean prioritizing those with no children on the grounds that childless patients would benefit more from the treatment than those with children, or it may mean prioritizing those *with* children if this can be taken as evidence for parenting ability.

There is a small body of evidence that having a first child is psychologically more important than having an additional child. This evidence is usefully summarized by Greil et al.,^39^ whose own study controlled for the numerous confounding factors in earlier studies, and came to the same conclusion that secondary infertility is associated with less distress than primary infertility. This may give us a patient‐welfare‐derived reason to prioritize those with no previous children. One participant's view reflected this: ‘[H]er need to have another child is not as high as that of somebody who hasn't got any children’ (09, p1).

Previous parenthood seemed to present a particular obstacle to receiving NHS‐funded treatment, where allocation of resources**—**this time purely financial**—**is an issue:

‘[T]he health service is short of money and you've already got one. So we'd prefer to put the money into some that's the way I would see it, into something, more important (01, p1).

One workshop participant further developed this way of prioritizing patients based on parental status, suggesting that if neither member of the couple has children, they should get the highest number of points; if only one member has children, fewer points; if both members have children, fewer still.

An alternative way of prioritizing based on parental status would be to prioritize those with proven parenting skills. Welfare of the child assessments may take into consideration a patient's track record regarding other, already‐existing children (for instance, if a child has been removed from the patient by social services this carries even greater weight against offering treatment than, say, existing children living with an individual). Indeed, some participants suggested that any record of violence to children, regardless of their relationship to the patient, could operate as a reason to deny a patient access to gametes. If it is the case that a *bad* track record with children can count *against* a patient for treatment, perhaps then a *good* track record should count *in favour of* a patient for treatment. Parental status may then be a marker for parenting capacity.

It would, however, be a mistake to draw this conclusion, because there is a significant asymmetry here. A person's having a history of abusing children speaks against them, in comparison to other patients, in a way that a person's having a history of being a good parent does not speak for them, in comparison to other patients. This is because in the latter case, those who have not had children have not yet had the chance to prove themselves as parents; all good parents were previously non‐parents.

The question of how ‘previous parenthood’ is defined is important,^40^ and participants offered a variety of views on this. That patients seeking fertility treatment are likely to view parenting experience as the goal rather than mere genetic reproduction (which they could perhaps achieve by donating gametes) suggests that the relevant conception of parenthood is one that incorporates raising children and having a relationship with them, rather than mere biological procreation.

One participant was adamant that previous children were only morally relevant if they were the genetic children of both partners. For this participant, even if a couple had a child living with them who was born from either member's previous relationship, this would not count against treatment:

Their own child. And as a couple together […]. If for example my partner has had children before is fairly irrelevant to me. It's either their own child, children before, or them as a couple. But not the male partner's previous children it's irrelevant. And that's quite often the situation if there is previous children, um where it will be the male partner's previous children. Second most common scenario would be that they as a couple together would have had one child perhaps before. I think maybe the female urge to have children is perhaps a bit stronger. I would give preference to that. Or that they want to have children together (10, p1)

It is not clear whether this participant meant that the child needed to be genetically related to them, or whether a child born from gamete donation would count as ‘their own child’. If genetic relatedness is necessary and sufficient for parenthood, then prioritization for treatment would only make sense in relation to couples or individuals using their *own* gametes. Anyone whose gametes are being replaced by donor gametes would be no closer to being a parent by having this treatment.

Another participant emphasized genetic relatedness, but did not comment on how this could affect those seeking treatment with donor gametes: [F]or couples that don't have their own genetic child, to be denied at least one go at NHS‐funded treatment is quite harsh (03, p1).^41^ Yet another, however, offered a different conception of parenthood based on the parenting experience, rather than genetic relatedness:

[I]f you have a, a couple has a child living with them, no matter how the child came about, if that's a criteria, even if child has left the home […] I would not want to offer them, ‘cos they have enjoyed bringing up a child. […]So they've had that experience. … It's better to give that opportunity to someone else who's never had that experience (15, p1)

Disagreement amongst participants about how to define parenthood resulted in a variety of views about how parental status should be used to determine allocation of donated gametes.

The spectrum of potential relationships with children is so large that coarse‐grained thresholds would be necessary for practical purposes. The approximate level of involvement that the patients have had with children, for example, may have to be represented by how much face‐to‐face contact they have with them.^42^ Further research could help to determine a consensus as to how close the relationship between a parent and their child needs to be before the parent is de‐prioritized, but we can still conclude that, all other things being equal, those who have enjoyed less experience of parenting should be given priority over those who have enjoyed more if, as we suggest, the goal of treatment is to achieve parenthood.

Some participants, however, felt that previous parenthood should play no part in allocation decisions, and this was often grounded in the idea that all patients are equal in their *desire* for treatment:

I don't think it matters if you've got one or two kids, if you want one more that's my experience (02, p1)

[I]t may be that having a second child is just as important if not more important than having a first child, or what however many children (04, p1)

I don't make any judgement about whether somebody has one child or 10 children, um, it's it's done on their desire. And I don't think it's right to judge whether we should allow patients to potentially have one, two or more children (06, p1)

One workshop participant wondered whether it is better to be an only child or to have siblings, and that it might be better to give a family another child rather than to create yet another family with only one child. As with participant 05 above, this frames the question of previous parenthood in terms of child welfare (both for the existing child *and* for the as yet unborn child) rather than in terms of patients' interests. It would provide a reason to favour those who *already have one child* over those who have none. Hence, the participants' comments show that even if we *can* account for previous parenthood, it is not clear whether it should cause us to prioritize or de‐prioritize that patient.

In summary, many participants opposed the introduction of parental status as a criterion for prioritizing, but other participants considered the nuances of how it might be implemented. Participants sometimes felt that patients who have already had this experience should not be offered treatment ahead of those who have not. On the other hand, one participant considered the view that the importance of having siblings may give us a reason to prioritize those with only one child over those with none. While welfare of the child considerations do not give us enough reason to distinguish between patients on the basis of whether they have siblings, considerations of patient welfare may do so insofar as primary infertility causes more psychological distress than secondary infertility.

### 3.3 Study limitations

The study did not include GPs or staff providing secondary level services (e.g., those at centres offering diagnostic testing for infertility). These individuals are also potential gatekeepers and may have identified different criteria for allocating gametes. Nor were current patients included even though it might be argued that their views on allocation are more relevant than those of healthcare professionals. We were not convinced that including current patients would have improved the quality of the resulting data sufficiently to outweigh the potential risks of appearing to challenge their reasons for wanting treatment, or the strength of their desire to have treatment sooner rather than later. Moreover, it seemed unlikely that patients would willingly explore positions that implied that people like them would be less likely to receive treatment, nor did we think it would be feasible to interview patients who had been excluded from treatment about the reasons behind this decision.

Interview participants were sometimes unable to offer considered responses to our questions in the limited time available to consider them in detail. With hindsight, pre‐circulating the questions may have enabled participants to give fuller, more considered responses.

## 4 CONCLUSIONS

There are good reasons to replace the FCFS system for allocating gametes with one where priority is given according to more morally relevant criteria. In this paper we have explored, as examples of such criteria: childlessness (although this concept requires careful definition); substance use (according to the type of substance and degree of use); and age (dependent on evidence around welfare and risk).

Our data suggest that replacing the FCFS system would meet with resistance that would need to be carefully addressed through more widespread and open debate on allocation criteria. For this reason, we have avoided commenting on the ordering or relative weight of these criteria, or how they interact (i.e., how points should be awarded), but have rather sought here to open up this hitherto largely neglected area for wider debate.

Prioritization/de‐prioritization is more ethically robust than placing patients on a waiting list and treating them in date order, so long as the criteria employed are relevant to the goals of providing treatment and do not unjustly discriminate. Of course, the use of criteria at all does indicate discrimination**—**but we would note that discrimination itself is acceptable if it is based on morally acceptable goals and morally relevant criteria. Prioritizing based on, for example, age and substance use seems fairly uncontroversial in principle, whereas the question of giving priority to patients who do not already have children is more problematic, given the possibility of more complicated cases such as those in which one member of a couple has children, or is actively engaged in parenting, and the other does/is not. This paper has suggested that all of these criteria can and should be accounted for when making treatment decisions (with the implication that others, not discussed here, may be relevant also), and that this system should replace the waiting list only system. This would lead to discrimination that is overall more just (assuming that it is evidence‐based and transparent) because it would factor in a range of morally relevant criteria, and would also lead to the most optimal outcomes for the most people. The criteria examined here are by no means exclusive, and it is important to note that the evidence needed to develop such a system, for all morally relevant criteria, might not yet exist. The argument presented does, however, serve to illustrate the potential justification for working towards a prioritization points system, which, whilst generating its own difficult question and debates, would replace a system that presents a facade of fairness simply through refusing to engage with the difficult questions.

## DATA STATEMENT

In line with the favourable opinion given by the ethics committee who reviewed this study, the dataset is not generally available. This is because the pool of people who were eligible to participate in the study was so small that releasing the full dataset would compromise the anonymity of our participants, as others may be able to identify participants if given access to full transcripts.

## Footnotes

1

Human Fertilisation and Embryology Authority . (2011). Donating sperm and eggs: Have your say. London, UK Retrieved from http://www.hfea.gov.uk/docs/2011-01-13_Donation_review_background.pdf

2
See, for example: Human Fertilisation and Embryology Act (1990). Retrieved from http://www.legislation.gov.uk/ukpga/1990/37; and its subsequent amendments: Human Fertilisation and Embryology Act (2008). Retrieved from http://www.legislation.gov.uk/ukpga/2008/22.

3
Ives, J., & Draper, H. (2009). Appropriate methodologies for empirical bioethics: It's all relative. *Bioethics*, *23*(4), 249–258; Musschenga, A. W. (2005). Empirical ethics, context‐sensitivity, and contextualism. *The Journal of Medicine and Philosophy*, *30*(5), 467–490. 10.1080/0360531050025303016282140

4
Ives & Draper, *op. cit*. note 3, p. 251.

5

Mill, J. S. (1991). On liberty and other essays. Oxford: Oxford University Press.

6

Huxtable, R. (2013). Law, ethics and compromise at the limits of life: To treat or not to treat? Abingdon: Routledge.

7

Ives, J. (2014). A method of reflexive balancing in a pragmatic, interdisciplinary and reflexive bioethics. Bioethics, 28(6), 302–312. 2344490910.1111/bioe.12018

8
In the UK, any research on NHS premises or with NHS staff requires permission from that Trust's research and development department.

9

Bryman, A. (2012). Social research methods (4th ed.). Oxford: Oxford University Press.

10
These participants' roles cannot be described in further detail due to the risk of incomplete anonymization, as some of them participated in phase 2.

11
Welsh, E. (2002). Dealing with data: Using Nvivo in the qualitative data analysis process. *Forum: Qualitative Social Research*, *3*(2), art. 26, 3.

12

Braun, V. , & Clarke, V. (2006). Using thematic analysis in psychology. Qualitative Research in Psychology, 3(2), 77–101.

13

Hsieh, H. , & Shannon, S. (2005). Three approaches to qualitative content analysis. Qualitative Health Research, 15, 1277–1288. 1620440510.1177/1049732305276687

14
In terms of traditional reporting methods, this means that the results and discussion have been combined rather than being presented as two discrete sections.

15
Pennings, G. (2001). Distributive justice in the allocation of donor oocytes. *Journal of Assisted Reproduction and Genetics*, *18*(2), 56–63, 57. 10.1023/A:1026570305919PMC345555811285981

16
Ibid: 57.

17
Farquhar, C. M., van den Boogaard, N. M., Riddell, C., Macdonald, A., Chan, E., & Mol, B. W. (2011). Accessing fertility treatment in New Zealand: A comparison of the clinical priority access criteria with a prediction model for couples with unexplained subfertility. *Human Reproduction, 26*(11), 3037–3044, 3038. 10.1093/humrep/der27921896547

18
Pennings, *op. cit* note 15; Peterson, M. M. (2005). Assisted reproductive technologies and equity of access issues. *Journal of Medical Ethics*, *31*(5), 280–285; ESHRE Task Force on Ethics and Law. (2010). Lifestyle‐related factors and access to medically assisted reproduction. *Human Reproduction*, *25*(3), 578–583; Pandey, S., Maheshwari, A., & Bhattacharya, S. (2010). Should access to fertility treatment be determined by female body mass index? *Human Reproduction,* 25(4), 815–820; Schon, S., & Butts, S. (2015). Obesity, reproductive outcomes, and access to infertility treatments: A clinical and ethical debate. In E. S. Jungheim (Ed.), *Obesity and fertility* (pp. 161–168). New York: Springer.

19
Robertson, J. A. (1994). *Children of choice: Freedom and the new reproductive technologies* Princeton, NJ: Princeton University Press; Cutas, D., & Bortolotti, L. (2010). Natural versus assisted reproduction: In search of fairness. *Studies in Ethics, Law and Technology*, *4*(1), 1–18.

20
LaFollette, H. (1980). Licensing parents. *Philosophy & Public Affairs*, *9*(2), 182–197; De Wispelaere, J., & Weinstock, D. (2014). State regulation and assisted reproduction: Balancing the interests of parents and children. In F. Baylis & C. McLeod (Eds.), *Family‐making: Contemporary ethical challenges* (pp. 131–150). New York: Oxford University Press.

21
Defined as the use of noxious substances that may harm a person or their children, or cause their future children to be worse off.

22
These numbers are the numbers assigned to the study participants.

23
Refers to study phase.

24
For more on treatment benefit versus equal treatment, see: Savulescu, J. (1998). Should doctors intentionally do less than the best? *Bioethics*, *12*(3), 212–235; Harris, J. (1999). Justice and equal opportunities in health care. *Bioethics*, *13*(5), 392–404. 10.1111/1467-8519.0016711658128

25
Caplan, A. L., & Patrizio, P. (2010). Are you ever too old to have a baby? The ethical challenges of older women using infertility services. *Seminars in Reproductive Medicine*, *28*(4), 281–286, 281. 10.1055/s-0030-125517520683791

26
General Medical Council. (2013). *Good medical practice* Retrieved from http://www.gmc-uk.org/guidance/good_medical_practice/duties_of_a_doctor.asp

27
Nursing and Midwifery Council. (2015). *The code* Retrieved from http://www.nmc-uk.org/Documents/NMC-Publications/revised-new-NMC-Code.pdf

28
Sauer, M. V., Paulson, R. J., & Lobo, R. A. (1996). Oocyte donation to women of advanced reproductive age: Pregnancy results and obstetrical outcomes in patients 45 years and older. *Human Reproduction*, *11*(11), 2540–2453; Abdalla, H. I., Wren, M. E., Thomas, A., & Korea, L. (1997). Age of the uterus does not affect pregnancy or implantation rates; a study of egg donation in women of different ages sharing oocytes from the same donor. *Human Reproduction*, *12*(4), 827–829; Paulson, R. J., Hatch, I. E., Lobo, R. A., & Sauer, M. V. (1997). Cumulative conception and live birth rates after oocyte donation: Implications regarding endometrial receptivity. *Human Reproduction*, *12*(4), 835–839; Noyes, N., Hampton, B. S., Berkeley, A., Licciardi, F., Grifo, J., & Krey, L. (2001). Factors useful in predicting the success of oocyte donation: A 3‐year retrospective analysis. *Fertility and Sterility*, *76*(1), 92–97.

29
Sauer et al., *op. cit*. note 28.

30
Sutcliffe, A. G., Barnes, J., Belsky, J., Gardiner, J., & Melhuish, E. (2012). The health and development of children born to older mothers in the United Kingdom: Observational study using longitudinal cohort data. *BMJ*, *345*, e5116. 10.1136/bmj.e5116PMC342422722915663

31
Ibid: 4.

32
Severity of the conditions for which admission was required was not recorded, however.

33
National Health Service. (2017). *Alcohol and drugs during pregnancy* Retrieved from http://www.nhs.uk/conditions/pregnancy-and-baby/pages/alcohol-medicines-drugs-pregnant.aspx; National Health Service. (2016). *Stop smoking in pregnancy* Retrieved from http://www.nhs.uk/conditions/pregnancy-and-baby/pages/smoking-pregnant.aspx; National Health Service. (2016). *Illegal drugs in pregnancy* Retrieved from http://www.nhs.uk/conditions/pregnancy-and-baby/pages/illegal-drugs-in-pregnancy.aspx

34
Human Fertilisation and Embryology Authority. (2009). *Code of practice* (8th ed.). Retrieved from http://www.hfea.gov.uk/docs/8th_Code_of_Practice_Upto102013.pdf

35
Taken here to mean illegal drugs.

36
Behnke, M., Smith, V. C., Committee on Substance Abuse, & Committee on Fetus and Newborn. (2013). Prenatal substance abuse: Short‐ and long‐term effects on the exposed fetus. *Pediatrics*, *131*(3), e1009–e1024. 10.1542/peds.2012-3931PMC819446423439891

37
Of course, it is also possible that “use of illegal drugs” may be used as proxy for judgement about (un)suitable character, on the assumption that engaging in illegal activity makes person less suitable as a parent. Discussion tended, however, to focus on the physiological risks of drug taking, and any concerns that may have linked drug taking to parental character went unarticulated.

38
Participant 16 may have meant “effective” instead of “accessible.”

39

Greil, A. L. , Shreffler, K. M. , Schmidt, L. , & McQuillan, J. (2011). Variation in distress among women with infertility: Evidence from a population‐based sample. Human Reproduction, 26(8), 2101–2112. 2165931310.1093/humrep/der148PMC3137388

40
For discussion on the definition and value of parenthood, see for example: Brighouse, H., & Swift, A. (2006). Parents' rights and the value of the family. *Ethics*, *117*(1), 80–108; Hall, B. (1999). The origin of parental rights. *Public Affairs Quarterly*, *13*(1), 73–82; Schoeman, F. (1980). Rights of children, rights of parents and the moral basis of the family. *Ethics*, *91*(1), 6–19.

41
It is possible that the participant was making a general point about NHS funding rather than a specific comment about allocating gametes.

42
Meaningful parenting might be difficult to achieve with no contact, but equally many hours of contact time does not necessarily equate to meaningful parenting. Likewise, some individuals have to maintain meaningful relationships with children using non‐face‐to‐face contact (phone, social media, texting, etc.).

